# Crystal structure of bis­(allyl­ammonium) oxalate

**DOI:** 10.1107/S1600536814023617

**Published:** 2014-11-05

**Authors:** Błażej Dziuk, Bartosz Zarychta, Krzysztof Ejsmont

**Affiliations:** a Faculty of Chemistry, University of Opole, Oleska 48, 45-052 Opole, Poland

**Keywords:** crystal structure, allyl­ammonium, oxalate, dication, hydrogen bonding

## Abstract

The title salt, 2C_3_H_8_N^+^·C_2_O_4_^2−^, crystallized with six independent allyl­ammonium cations and three independent oxalate dianions in the asymmetric unit. One of the oxalate dianions is nearly planar [dihedral angle between CO_2_ planes = 1.91 (19)°], while the other two are twisted with angles of 11.3 (3) and 26.09 (13)°. One cation has a synperiplanar (*cis*) conformation with an N—C—C—C torsion angle of 0.9 (3)°, whereas the five remaining cations are characterized by *gauche* arrangements, with the N—C—C—C torsion angles ranging from 115.9 (12) to 128.8 (3)°. One of the allyl­ammonium cations is positionally disordered (fixed occupancy ratio = 0.45:0.55). In the crystal, the cations and anions are connected by a number of strong N—H⋯O and N—H⋯(O,O) hydrogen bonds, forming layers parallel to (001), with the vinyl groups protruding into the space between the layers.

## Related literature   

For the crystal structures of oxalic acid salts with aliphatic amines, see: Dziuk *et al.* (2014*a* ▶,*b* ▶); Braga *et al.* (2013 ▶); Ejsmont & Zaleski (2006*a* ▶,*b* ▶); Ejsmont (2006 ▶, 2007 ▶). For the crystal structures of salts with disordered allyl­ammonium cations, see: Płowaś *et al.* (2010 ▶); Zarychta *et al.* (2007 ▶). 
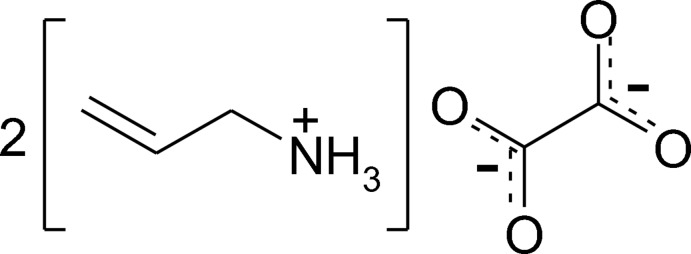


## Experimental   

### Crystal data  

2C_3_H_8_N^+^·C_2_O_4_^−^
*M* *_r_* = 204.23
Monoclinic, 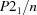

*a* = 6.7060 (3) Å
*b* = 12.1364 (10) Å
*c* = 40.6017 (16) Å
β = 93.969 (4)°
*V* = 3296.5 (3) Å^3^
*Z* = 12
Mo *K*α radiation
μ = 0.10 mm^−1^
*T* = 100 K
0.25 × 0.15 × 0.10 mm

### Data collection  

Oxford Diffraction Xcalibur CCD diffractometer
21821 measured reflections
6460 independent reflections
4306 reflections with *I* > 2σ(*I*)
*R*_int_ = 0.036

### Refinement  

*R*[*F* ^2^ > 2σ(*F* ^2^)] = 0.053
*wR*(*F* ^2^) = 0.132
*S* = 1.03
6460 reflections
397 parameters
38 restraints
H-atom parameters constrained
Δρ_max_ = 0.57 e Å^−3^
Δρ_min_ = −0.44 e Å^−3^

### 

Data collection: *CrysAlis CCD* (Oxford Diffraction, 2008 ▶); cell refinement: *CrysAlis CCD*; data reduction: *CrysAlis RED* (Oxford Diffraction, 2008 ▶); program(s) used to solve structure: *SHELXS2013* (Sheldrick, 2008 ▶); program(s) used to refine structure: *SHELXL2013* (Sheldrick, 2008 ▶); molecular graphics: *SHELXTL* (Sheldrick, 2008 ▶) and *PLATON* (Spek, 2009 ▶); software used to prepare material for publication: *SHELXL2013*.

## Supplementary Material

Crystal structure: contains datablock(s) global, I. DOI: 10.1107/S1600536814023617/su5011sup1.cif

Structure factors: contains datablock(s) I. DOI: 10.1107/S1600536814023617/su5011Isup2.hkl

Supporting information file. DOI: 10.1107/S1600536814023617/su5011Isup3.cml

**Fig. 1.**
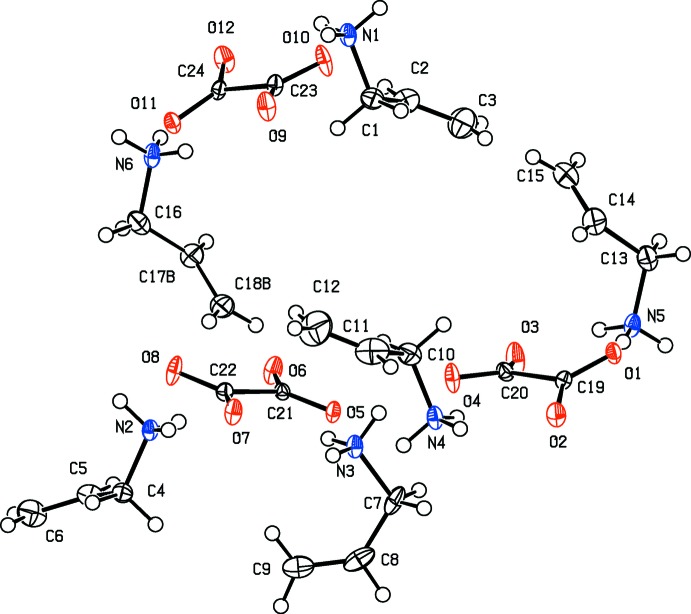
The molecular structure of the asymmetric unit of the title salt, with atom labelling. Displacement ellipsoids are drawn at the 50% probability level. One of the allylammonium cations is positionally disorded and only the major components, atoms C17B and C18B, are shown for clarity.

**Fig. 2.**
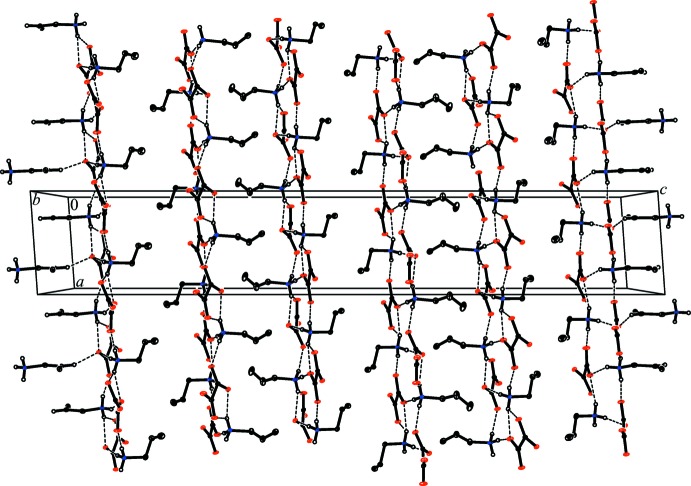
The crystal packing viewed along the b axis of the title salt. The hydrogen bonds are shown as dashed lines (see Table 1 for details).

B B . DOI: 10.1107/S1600536814023617/su5011fig1.tif
The mol­ecular structure of the asymmetric unit of the title salt, with atom labelling. Displacement ellipsoids are drawn at the 50% probability level. One of the allyl­ammonium cations is positionally disorded and only the major components, atoms C17*B* and C18*B*, are shown for clarity.

b . DOI: 10.1107/S1600536814023617/su5011fig2.tif
The crystal packing viewed along the *b* axis of the title salt. The hydrogen bonds are shown as dashed lines (see Table 1 for details).

CCDC reference: 1031212

Additional supporting information:  crystallographic information; 3D view; checkCIF report

## Figures and Tables

**Table 1:** Hydrogen-bond geometry (, )

*D*H*A*	*D*H	H*A*	*D* *A*	*D*H*A*
N1H1*A*O10	0.89	2.02	2.829(2)	150
N1H1*A*O12	0.89	2.30	2.905(2)	125
N1H1*B*O9^i^	0.89	1.89	2.768(2)	167
N1H1*C*O11^ii^	0.89	1.92	2.801(2)	169
N2H2*A*O6^iii^	0.89	2.32	2.890(2)	122
N2H2*A*O8^iii^	0.89	2.02	2.838(2)	152
N2H2*B*O1^iv^	0.89	1.92	2.797(2)	168
N2H2*C*O7	0.89	1.89	2.773(2)	172
N3H3*A*O4	0.89	1.94	2.737(2)	148
N3H3*B*O6^iii^	0.89	1.89	2.759(2)	165
N3H3*C*O5	0.89	2.12	2.847(2)	138
N3H3*C*O7	0.89	2.13	2.885(2)	142
N4H4*A*O2	0.89	2.18	2.887(2)	135
N4H4*A*O4	0.89	2.10	2.831(2)	139
N4H4*B*O5^iii^	0.89	1.89	2.769(2)	172
N4H4*C*O3^iii^	0.89	1.84	2.728(2)	176
N5H5*A*O8^v^	0.89	1.83	2.701(2)	165
N5H5*B*O2^i^	0.89	1.93	2.762(2)	156
N5H5*C*O1	0.89	2.09	2.945(2)	162
N6H6*A*O10^vi^	0.89	1.85	2.720(2)	166
N6H6*B*O11	0.89	2.04	2.900(2)	163
N6H6*C*O12^iii^	0.89	1.92	2.744(2)	153

Symmetry codes: (i) 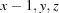; (ii) 
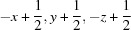
; (iii) 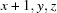; (iv) 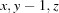; (v) 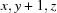; (vi) 
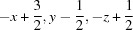
.

## supplementary crystallographic information

### S1. Comment

The crystal structure of the title salt is illustrated in Fig. 1. It crystallized with six independent allylammonium cations and three independent oxalate dianions in the asymmetric unit. This is in contrast to the structure of a ethylammonium oxalate hemihydrate where the oxalate is present as a monoanion (Ejsmont & Zaleski, 2006a).

The geometry of the anions and cations is alternated. One of oxalate anion is nearly planar with the O5–C21–C22–O8 torsion angle being 179.22 (19)°, while the two remaining oxalate anions are twisted along the C19–C20 and C23–C24 bonds by -154.87 (19)° and 168.12 (19)°. The N3 cation has a *syn*-periplanar (*cis*) conformation with the N–C–C–C torsion angle of 0.9 (3)°, whereas the five remaining cations are characterized by a *gauche* arrangement, with torsion angles ranging from 115.9 (12) to 128.8 (3)° for the N6 and N4 cations, respectively.

Moreover one allylammonium cation is disordered, with a similar type of disorder as in the structures of (C_3_H_5_NH_3_)_3_ [SbBr_6_] (Płowaś *et al.*, 2010) and (C_3_H_5_NH_3_)_2_SbCl_5_^.^(C_3_H_5_NH_3_)Cl (Zarychta *et al.*, 2007).

In the crystal, each anion is hydrogen bonded to two cations via N-H···O hydrogen bonds (Table 1 and Fig. 2) forming layers parallel to (001), separated by ca. 7.9 Å. The space is occupied by the vinyl groups of the allylammonium cations (Fig. 2 and Table 1).

### S2. Experimental

Crystals were grown at room temperature by slow evaporation of an aqueous solution containing allylamine and oxalic acid in a 1:1 stoichiometric ratio.

### S3. Refinement

All H atoms were positioned geometrically and treated as riding on their parent atoms, with N–H = 0.89 Å, C—H = 0.93 - 0.97 Å and with *U*_iso_(H) = 1.5*U*_eq_(*N*,*C*-methyl) and = 1.2*U*_eq_(C) for other H atoms. One of the allylammonium cations is positionally disorded and atoms C17A/C17B and C18A/C18B were refined with a fixed occupany ratio of 0.45:0.55.

### Crystal data

**Table:**

2C_3_H_8_N^+^·C_2_O_4_^−^	*F*(000) = 1320
*M**_r_* = 204.23	*D*_x_ = 1.235 Mg m^−^^3^
Monoclinic, *P*2_1_/*n*	Mo *K*α radiation, λ = 0.71073 Å
*a* = 6.7060 (3) Å	Cell parameters from 21821 reflections
*b* = 12.1364 (10) Å	θ = 3.0–26.0°
*c* = 40.6017 (16) Å	µ = 0.10 mm^−^^1^
β = 93.969 (4)°	*T* = 100 K
*V* = 3296.5 (3) Å^3^	Block, colourless
*Z* = 12	0.25 × 0.15 × 0.10 mm

### Data collection

**Table:**

Oxford Diffraction Xcalibur CCD diffractometer	4306 reflections with *I* > 2σ(*I*)
Radiation source: fine-focus sealed tube	*R*_int_ = 0.036
Graphite monochromator	θ_max_ = 26.0°, θ_min_ = 3.0°
ω scan	*h* = −8→8
21821 measured reflections	*k* = −14→13
6460 independent reflections	*l* = −49→47

### Refinement

**Table:**

Refinement on *F*^2^	38 restraints
Least-squares matrix: full	Hydrogen site location: inferred from neighbouring sites
*R*[*F*^2^ > 2σ(*F*^2^)] = 0.053	H-atom parameters constrained
*wR*(*F*^2^) = 0.132	*w* = 1/[σ^2^(*F*_o_^2^) + (0.0677*P*)^2^] where *P* = (*F*_o_^2^ + 2*F*_c_^2^)/3
*S* = 1.03	(Δ/σ)_max_ < 0.001
6460 reflections	Δρ_max_ = 0.57 e Å^−^^3^
397 parameters	Δρ_min_ = −0.44 e Å^−^^3^

### Special details

**Table:**

Geometry. All e.s.d.'s (except the e.s.d. in the dihedral angle between two l.s. planes) are estimated using the full covariance matrix. The cell e.s.d.'s are taken into account individually in the estimation of e.s.d.'s in distances, angles and torsion angles; correlations between e.s.d.'s in cell parameters are only used when they are defined by crystal symmetry. An approximate (isotropic) treatment of cell e.s.d.'s is used for estimating e.s.d.'s involving l.s. planes.

### Fractional atomic coordinates and isotropic or equivalent isotropic displacement parameters (Å^2^)

**Table:**

	*x*	*y*	*z*	*U*_iso_*/*U*_eq_	Occ. (<1)
N1	0.0693 (2)	0.39719 (14)	0.24556 (4)	0.0168 (4)	
H1A	0.1840	0.3626	0.2509	0.025*	
H1B	−0.0319	0.3503	0.2469	0.025*	
H1C	0.0549	0.4530	0.2594	0.025*	
C1	0.0706 (3)	0.43979 (19)	0.21148 (5)	0.0233 (5)	
H1D	0.0888	0.3789	0.1965	0.028*	
H1E	0.1825	0.4898	0.2101	0.028*	
C2	−0.1179 (4)	0.4984 (2)	0.20118 (6)	0.0286 (6)	
H2D	−0.2376	0.4615	0.2035	0.034*	
C3	−0.1275 (4)	0.5981 (2)	0.18910 (7)	0.0423 (7)	
H3D	−0.0108	0.6375	0.1864	0.051*	
H3E	−0.2511	0.6297	0.1832	0.051*	
N2	0.9169 (2)	0.04985 (13)	0.07308 (4)	0.0146 (4)	
H2A	1.0339	0.0818	0.0786	0.022*	
H2B	0.8945	−0.0030	0.0876	0.022*	
H2C	0.8199	0.0999	0.0731	0.022*	
C4	0.9211 (3)	0.00129 (18)	0.03964 (5)	0.0197 (5)	
H4D	1.0297	−0.0514	0.0395	0.024*	
H4E	0.9463	0.0591	0.0239	0.024*	
C5	0.7292 (3)	−0.0550 (2)	0.02916 (6)	0.0247 (5)	
H5D	0.6114	−0.0148	0.0298	0.030*	
C6	0.7165 (4)	−0.1577 (2)	0.01908 (6)	0.0354 (7)	
H6D	0.8317	−0.1999	0.0182	0.042*	
H6E	0.5923	−0.1882	0.0129	0.042*	
N3	0.7745 (2)	0.40896 (13)	0.05764 (4)	0.0159 (4)	
H3A	0.7731	0.4511	0.0756	0.024*	
H3B	0.8881	0.3709	0.0582	0.024*	
H3C	0.6716	0.3625	0.0571	0.024*	
C7	0.7589 (3)	0.47910 (18)	0.02790 (6)	0.0262 (6)	
H7A	0.8698	0.5306	0.0290	0.031*	
H7B	0.6364	0.5216	0.0278	0.031*	
C8	0.7597 (3)	0.4167 (2)	−0.00353 (6)	0.0303 (6)	
H8A	0.7520	0.4582	−0.0228	0.036*	
C9	0.7701 (4)	0.3098 (2)	−0.00713 (6)	0.0319 (6)	
H9A	0.7781	0.2642	0.0113	0.038*	
H9B	0.7694	0.2793	−0.0282	0.038*	
N4	1.2953 (2)	0.54981 (14)	0.09731 (4)	0.0173 (4)	
H4A	1.1841	0.5880	0.0923	0.026*	
H4B	1.3041	0.4947	0.0830	0.026*	
H4C	1.4009	0.5939	0.0963	0.026*	
C10	1.2902 (4)	0.5047 (2)	0.13118 (6)	0.0285 (6)	
H10A	1.2791	0.5650	0.1466	0.034*	
H10B	1.1728	0.4585	0.1323	0.034*	
C11	1.4686 (4)	0.4398 (2)	0.14102 (6)	0.0382 (7)	
H11A	1.5926	0.4719	0.1385	0.046*	
C12	1.4659 (5)	0.3402 (3)	0.15312 (7)	0.0548 (9)	
H12A	1.3446	0.3057	0.1560	0.066*	
H12B	1.5854	0.3038	0.1588	0.066*	
N5	0.4296 (2)	0.89624 (14)	0.10655 (4)	0.0174 (4)	
H5A	0.3922	0.9540	0.0941	0.026*	
H5B	0.3529	0.8386	0.1007	0.026*	
H5C	0.5567	0.8801	0.1037	0.026*	
C13	0.4079 (4)	0.92265 (19)	0.14159 (6)	0.0270 (6)	
H13A	0.4922	0.9853	0.1478	0.032*	
H13B	0.2705	0.9436	0.1443	0.032*	
C14	0.4622 (5)	0.8297 (2)	0.16383 (7)	0.0433 (7)	
H14A	0.5926	0.8043	0.1630	0.052*	
C15	0.3600 (5)	0.7797 (2)	0.18369 (7)	0.0518 (9)	
H15A	0.2281	0.8004	0.1859	0.062*	
H15B	0.4159	0.7219	0.1962	0.062*	
N6	0.9227 (2)	0.05348 (14)	0.22058 (4)	0.0177 (4)	
H6A	0.9646	−0.0048	0.2325	0.027*	
H6B	0.7959	0.0677	0.2243	0.027*	
H6C	0.9981	0.1116	0.2264	0.027*	
C16	0.9382 (4)	0.0301 (2)	0.18525 (6)	0.0298 (6)	
H16A	1.0365	−0.0278	0.1833	0.036*	
H16B	0.8106	0.0016	0.1763	0.036*	
C17A	0.9923 (12)	0.1216 (5)	0.16504 (16)	0.0347 (15)	0.45
H17A	1.1199	0.1483	0.1709	0.042*	0.45
C18A	0.9094 (14)	0.1719 (13)	0.1421 (4)	0.038 (3)	0.45
H18A	0.7810	0.1521	0.1342	0.045*	0.45
H18B	0.9745	0.2300	0.1325	0.045*	0.45
C17B	0.8712 (8)	0.1232 (4)	0.16450 (13)	0.0240 (11)	0.55
H17B	0.7410	0.1482	0.1660	0.029*	0.55
C18B	0.9883 (12)	0.1764 (10)	0.1429 (3)	0.033 (2)	0.55
H18C	1.1192	0.1534	0.1408	0.040*	0.55
H18D	0.9364	0.2352	0.1303	0.040*	0.55
O1	0.8657 (2)	0.86307 (11)	0.11197 (4)	0.0174 (3)	
O2	1.1046 (2)	0.76253 (12)	0.09007 (4)	0.0212 (4)	
O3	0.6117 (2)	0.69093 (12)	0.09661 (4)	0.0297 (4)	
O4	0.8778 (2)	0.58025 (11)	0.09925 (4)	0.0239 (4)	
C19	0.9343 (3)	0.77562 (17)	0.10068 (5)	0.0143 (5)	
C20	0.7955 (3)	0.67310 (17)	0.09876 (5)	0.0162 (5)	
O5	0.3551 (2)	0.37126 (11)	0.05668 (3)	0.0159 (3)	
O6	0.0928 (2)	0.26559 (12)	0.06678 (4)	0.0222 (4)	
O7	0.5985 (2)	0.19598 (11)	0.06801 (4)	0.0191 (4)	
O8	0.3342 (2)	0.09181 (11)	0.07784 (4)	0.0219 (4)	
C21	0.2742 (3)	0.28163 (16)	0.06386 (5)	0.0122 (4)	
C22	0.4160 (3)	0.18068 (16)	0.07032 (5)	0.0139 (5)	
O9	0.7477 (2)	0.25409 (12)	0.23883 (4)	0.0237 (4)	
O10	0.4852 (2)	0.35506 (12)	0.25117 (4)	0.0257 (4)	
O11	0.4942 (2)	0.08883 (11)	0.21818 (4)	0.0173 (3)	
O12	0.2475 (2)	0.18011 (12)	0.24079 (4)	0.0226 (4)	
C23	0.5651 (3)	0.26894 (17)	0.24167 (5)	0.0159 (5)	
C24	0.4224 (3)	0.17033 (16)	0.23246 (5)	0.0145 (5)	

### Atomic displacement parameters (Å^2^)

**Table:**

	*U*^11^	*U*^22^	*U*^33^	*U*^12^	*U*^13^	*U*^23^
N1	0.0115 (9)	0.0126 (9)	0.0267 (11)	0.0018 (7)	0.0032 (8)	−0.0010 (8)
C1	0.0230 (13)	0.0245 (13)	0.0229 (13)	0.0013 (10)	0.0052 (10)	−0.0006 (10)
C2	0.0228 (13)	0.0355 (15)	0.0271 (14)	−0.0015 (11)	−0.0021 (11)	0.0036 (12)
C3	0.0470 (18)	0.0395 (17)	0.0396 (17)	0.0115 (14)	−0.0023 (14)	0.0070 (14)
N2	0.0111 (9)	0.0115 (9)	0.0215 (10)	−0.0007 (7)	0.0030 (7)	0.0005 (8)
C4	0.0203 (12)	0.0191 (12)	0.0203 (13)	0.0009 (9)	0.0057 (9)	−0.0006 (9)
C5	0.0182 (12)	0.0328 (14)	0.0228 (13)	0.0016 (10)	0.0006 (10)	−0.0030 (11)
C6	0.0305 (15)	0.0404 (17)	0.0354 (16)	−0.0147 (12)	0.0043 (12)	−0.0091 (13)
N3	0.0099 (9)	0.0121 (9)	0.0258 (11)	0.0013 (7)	0.0019 (7)	0.0002 (8)
C7	0.0200 (13)	0.0198 (12)	0.0389 (15)	0.0038 (10)	0.0029 (11)	0.0156 (11)
C8	0.0174 (13)	0.0453 (17)	0.0284 (15)	0.0011 (11)	0.0036 (10)	0.0180 (12)
C9	0.0280 (15)	0.0460 (17)	0.0215 (14)	0.0002 (12)	0.0008 (11)	0.0024 (12)
N4	0.0132 (9)	0.0116 (9)	0.0271 (11)	0.0004 (7)	0.0028 (8)	−0.0016 (8)
C10	0.0341 (15)	0.0277 (14)	0.0243 (14)	0.0049 (11)	0.0064 (11)	−0.0018 (11)
C11	0.0286 (15)	0.0543 (19)	0.0316 (16)	0.0057 (13)	0.0012 (11)	0.0025 (14)
C12	0.051 (2)	0.051 (2)	0.063 (2)	0.0295 (16)	0.0130 (16)	0.0173 (17)
N5	0.0120 (9)	0.0124 (9)	0.0284 (11)	0.0037 (7)	0.0052 (8)	0.0034 (8)
C13	0.0320 (14)	0.0199 (13)	0.0291 (14)	0.0025 (10)	0.0023 (11)	−0.0026 (11)
C14	0.071 (2)	0.0266 (15)	0.0324 (16)	0.0002 (14)	0.0049 (15)	−0.0014 (13)
C15	0.089 (3)	0.0298 (16)	0.0397 (18)	0.0032 (16)	0.0267 (17)	−0.0064 (14)
N6	0.0115 (9)	0.0122 (9)	0.0298 (11)	0.0018 (7)	0.0038 (8)	0.0012 (8)
C16	0.0402 (16)	0.0240 (14)	0.0251 (14)	0.0026 (11)	0.0022 (11)	−0.0071 (11)
C17A	0.044 (4)	0.034 (3)	0.027 (3)	−0.002 (3)	0.007 (3)	−0.004 (3)
C18A	0.035 (6)	0.041 (5)	0.038 (4)	0.004 (6)	0.010 (6)	−0.003 (4)
C17B	0.020 (3)	0.025 (2)	0.026 (3)	0.002 (2)	0.001 (2)	0.002 (2)
C18B	0.044 (5)	0.027 (3)	0.030 (4)	−0.010 (5)	0.014 (5)	−0.003 (3)
O1	0.0182 (8)	0.0107 (8)	0.0238 (9)	0.0014 (6)	0.0054 (7)	−0.0017 (6)
O2	0.0151 (8)	0.0170 (8)	0.0322 (9)	−0.0004 (6)	0.0075 (7)	−0.0012 (7)
O3	0.0121 (9)	0.0157 (8)	0.0617 (13)	0.0009 (6)	0.0045 (8)	−0.0035 (8)
O4	0.0193 (8)	0.0096 (8)	0.0430 (11)	0.0015 (6)	0.0030 (7)	−0.0033 (7)
C19	0.0149 (11)	0.0124 (11)	0.0155 (11)	0.0007 (9)	0.0006 (9)	0.0017 (9)
C20	0.0163 (12)	0.0142 (11)	0.0187 (12)	−0.0004 (9)	0.0054 (9)	−0.0032 (9)
O5	0.0149 (8)	0.0095 (7)	0.0236 (9)	−0.0001 (6)	0.0045 (6)	0.0020 (6)
O6	0.0112 (8)	0.0159 (8)	0.0401 (10)	0.0028 (6)	0.0049 (7)	0.0070 (7)
O7	0.0100 (8)	0.0136 (8)	0.0340 (10)	0.0015 (6)	0.0036 (6)	0.0030 (7)
O8	0.0120 (8)	0.0114 (8)	0.0425 (10)	0.0013 (6)	0.0026 (7)	0.0098 (7)
C21	0.0117 (11)	0.0111 (10)	0.0141 (11)	0.0016 (8)	0.0019 (8)	−0.0007 (8)
C22	0.0120 (11)	0.0133 (11)	0.0165 (11)	−0.0002 (9)	0.0022 (8)	0.0005 (9)
O9	0.0123 (8)	0.0156 (8)	0.0436 (11)	−0.0008 (6)	0.0054 (7)	−0.0042 (7)
O10	0.0167 (9)	0.0126 (8)	0.0479 (11)	0.0013 (6)	0.0039 (7)	−0.0110 (7)
O11	0.0171 (8)	0.0126 (8)	0.0227 (9)	0.0005 (6)	0.0041 (6)	−0.0034 (6)
O12	0.0118 (8)	0.0193 (8)	0.0376 (10)	−0.0019 (6)	0.0072 (7)	−0.0065 (7)
C23	0.0142 (11)	0.0118 (11)	0.0217 (12)	0.0005 (9)	0.0013 (9)	0.0008 (9)
C24	0.0145 (12)	0.0103 (11)	0.0188 (12)	0.0010 (9)	0.0025 (9)	0.0026 (9)

### Geometric parameters (Å, º)

**Table:**

N1—C1	1.478 (3)	C12—H12B	0.9300
N1—H1A	0.8900	N5—C13	1.475 (3)
N1—H1B	0.8900	N5—H5A	0.8900
N1—H1C	0.8900	N5—H5B	0.8900
C1—C2	1.485 (3)	N5—H5C	0.8900
C1—H1D	0.9700	C13—C14	1.475 (3)
C1—H1E	0.9700	C13—H13A	0.9700
C2—C3	1.305 (3)	C13—H13B	0.9700
C2—H2D	0.9300	C14—C15	1.251 (4)
C3—H3D	0.9300	C14—H14A	0.9300
C3—H3E	0.9300	C15—H15A	0.9300
N2—C4	1.482 (3)	C15—H15B	0.9300
N2—H2A	0.8900	N6—C16	1.473 (3)
N2—H2B	0.8900	N6—H6A	0.8900
N2—H2C	0.8900	N6—H6B	0.8900
C4—C5	1.492 (3)	N6—H6C	0.8900
C4—H4D	0.9700	C16—C17A	1.442 (7)
C4—H4E	0.9700	C16—C17B	1.461 (6)
C5—C6	1.313 (3)	C16—H16A	0.9700
C5—H5D	0.9300	C16—H16B	0.9700
C6—H6D	0.9300	C17A—C18A	1.215 (17)
C6—H6E	0.9300	C17A—H17A	0.9300
N3—C7	1.475 (3)	C18A—H18A	0.9300
N3—H3A	0.8900	C18A—H18B	0.9300
N3—H3B	0.8900	C17B—C18B	1.379 (11)
N3—H3C	0.8900	C17B—H17B	0.9300
C7—C8	1.484 (3)	C18B—H18C	0.9300
C7—H7A	0.9700	C18B—H18D	0.9300
C7—H7B	0.9700	O1—C19	1.256 (2)
C8—C9	1.308 (3)	O2—C19	1.259 (2)
C8—H8A	0.9300	O3—C20	1.249 (2)
C9—H9A	0.9300	O4—C20	1.254 (2)
C9—H9B	0.9300	C19—C20	1.552 (3)
N4—C10	1.483 (3)	O5—C21	1.259 (2)
N4—H4A	0.8900	O6—C21	1.246 (2)
N4—H4B	0.8900	O7—C22	1.247 (2)
N4—H4C	0.8900	O8—C22	1.257 (2)
C10—C11	1.464 (3)	C21—C22	1.562 (3)
C10—H10A	0.9700	O9—C23	1.251 (2)
C10—H10B	0.9700	O10—C23	1.248 (2)
C11—C12	1.305 (4)	O11—C24	1.259 (2)
C11—H11A	0.9300	O12—C24	1.249 (2)
C12—H12A	0.9300	C23—C24	1.562 (3)
			
C1—N1—H1A	109.5	C12—C11—H11A	117.7
C1—N1—H1B	109.5	C10—C11—H11A	117.7
H1A—N1—H1B	109.5	C11—C12—H12A	120.0
C1—N1—H1C	109.5	C11—C12—H12B	120.0
H1A—N1—H1C	109.5	H12A—C12—H12B	120.0
H1B—N1—H1C	109.5	C13—N5—H5A	109.5
N1—C1—C2	111.78 (18)	C13—N5—H5B	109.5
N1—C1—H1D	109.3	H5A—N5—H5B	109.5
C2—C1—H1D	109.3	C13—N5—H5C	109.5
N1—C1—H1E	109.3	H5A—N5—H5C	109.5
C2—C1—H1E	109.3	H5B—N5—H5C	109.5
H1D—C1—H1E	107.9	C14—C13—N5	112.82 (19)
C3—C2—C1	124.7 (2)	C14—C13—H13A	109.0
C3—C2—H2D	117.7	N5—C13—H13A	109.0
C1—C2—H2D	117.7	C14—C13—H13B	109.0
C2—C3—H3D	120.0	N5—C13—H13B	109.0
C2—C3—H3E	120.0	H13A—C13—H13B	107.8
H3D—C3—H3E	120.0	C15—C14—C13	130.0 (3)
C4—N2—H2A	109.5	C15—C14—H14A	115.0
C4—N2—H2B	109.5	C13—C14—H14A	115.0
H2A—N2—H2B	109.5	C14—C15—H15A	120.0
C4—N2—H2C	109.5	C14—C15—H15B	120.0
H2A—N2—H2C	109.5	H15A—C15—H15B	120.0
H2B—N2—H2C	109.5	C16—N6—H6A	109.5
N2—C4—C5	111.87 (17)	C16—N6—H6B	109.5
N2—C4—H4D	109.2	H6A—N6—H6B	109.5
C5—C4—H4D	109.2	C16—N6—H6C	109.5
N2—C4—H4E	109.2	H6A—N6—H6C	109.5
C5—C4—H4E	109.2	H6B—N6—H6C	109.5
H4D—C4—H4E	107.9	C17A—C16—N6	116.3 (3)
C6—C5—C4	124.0 (2)	C17B—C16—N6	111.9 (3)
C6—C5—H5D	118.0	C17A—C16—H16A	108.2
C4—C5—H5D	118.0	N6—C16—H16A	108.2
C5—C6—H6D	120.0	C17A—C16—H16B	108.2
C5—C6—H6E	120.0	N6—C16—H16B	108.2
H6D—C6—H6E	120.0	H16A—C16—H16B	107.4
C7—N3—H3A	109.5	C18A—C17A—C16	134.7 (9)
C7—N3—H3B	109.5	C18A—C17A—H17A	112.7
H3A—N3—H3B	109.5	C16—C17A—H17A	112.7
C7—N3—H3C	109.5	C17A—C18A—H18A	120.0
H3A—N3—H3C	109.5	C17A—C18A—H18B	120.0
H3B—N3—H3C	109.5	H18A—C18A—H18B	120.0
N3—C7—C8	113.89 (19)	C18B—C17B—C16	124.3 (6)
N3—C7—H7A	108.8	C18B—C17B—H17B	117.9
C8—C7—H7A	108.8	C16—C17B—H17B	117.9
N3—C7—H7B	108.8	C17B—C18B—H18C	120.0
C8—C7—H7B	108.8	C17B—C18B—H18D	120.0
H7A—C7—H7B	107.7	H18C—C18B—H18D	120.0
C9—C8—C7	127.3 (2)	O1—C19—O2	126.54 (19)
C9—C8—H8A	116.4	O1—C19—C20	117.53 (18)
C7—C8—H8A	116.4	O2—C19—C20	115.91 (18)
C8—C9—H9A	120.0	O3—C20—O4	126.0 (2)
C8—C9—H9B	120.0	O3—C20—C19	116.75 (18)
H9A—C9—H9B	120.0	O4—C20—C19	117.23 (18)
C10—N4—H4A	109.5	O6—C21—O5	126.61 (19)
C10—N4—H4B	109.5	O6—C21—C22	116.67 (17)
H4A—N4—H4B	109.5	O5—C21—C22	116.71 (17)
C10—N4—H4C	109.5	O7—C22—O8	126.35 (19)
H4A—N4—H4C	109.5	O7—C22—C21	117.36 (17)
H4B—N4—H4C	109.5	O8—C22—C21	116.28 (18)
C11—C10—N4	112.4 (2)	O10—C23—O9	126.4 (2)
C11—C10—H10A	109.1	O10—C23—C24	116.53 (18)
N4—C10—H10A	109.1	O9—C23—C24	117.08 (18)
C11—C10—H10B	109.1	O12—C24—O11	126.59 (19)
N4—C10—H10B	109.1	O12—C24—C23	115.73 (18)
H10A—C10—H10B	107.9	O11—C24—C23	117.67 (18)
C12—C11—C10	124.6 (3)		
			
N1—C1—C2—C3	127.2 (3)	O1—C19—C20—O4	−154.87 (19)
N2—C4—C5—C6	−125.8 (2)	O2—C19—C20—O4	26.3 (3)
N3—C7—C8—C9	0.9 (3)	O6—C21—C22—O7	−178.86 (18)
N4—C10—C11—C12	−128.8 (3)	O5—C21—C22—O7	0.2 (3)
N5—C13—C14—C15	−120.8 (3)	O6—C21—C22—O8	0.2 (3)
C17B—C16—C17A—C18A	−26.1 (11)	O5—C21—C22—O8	179.22 (19)
N6—C16—C17A—C18A	−115.9 (12)	O10—C23—C24—O12	−11.9 (3)
C17A—C16—C17B—C18B	16.7 (7)	O9—C23—C24—O12	168.12 (19)
N6—C16—C17B—C18B	121.6 (7)	O10—C23—C24—O11	169.54 (19)
O1—C19—C20—O3	25.7 (3)	O9—C23—C24—O11	−10.5 (3)
O2—C19—C20—O3	−153.11 (19)		

### Hydrogen-bond geometry (Å, º)

**Table:**

*D*—H···*A*	*D*—H	H···*A*	*D*···*A*	*D*—H···*A*
N1—H1*A*···O10	0.89	2.02	2.829 (2)	150
N1—H1*A*···O12	0.89	2.30	2.905 (2)	125
N1—H1*B*···O9^i^	0.89	1.89	2.768 (2)	167
N1—H1*C*···O11^ii^	0.89	1.92	2.801 (2)	169
N2—H2*A*···O6^iii^	0.89	2.32	2.890 (2)	122
N2—H2*A*···O8^iii^	0.89	2.02	2.838 (2)	152
N2—H2*B*···O1^iv^	0.89	1.92	2.797 (2)	168
N2—H2*C*···O7	0.89	1.89	2.773 (2)	172
N3—H3*A*···O4	0.89	1.94	2.737 (2)	148
N3—H3*B*···O6^iii^	0.89	1.89	2.759 (2)	165
N3—H3*C*···O5	0.89	2.12	2.847 (2)	138
N3—H3*C*···O7	0.89	2.13	2.885 (2)	142
N4—H4*A*···O2	0.89	2.18	2.887 (2)	135
N4—H4*A*···O4	0.89	2.10	2.831 (2)	139
N4—H4*B*···O5^iii^	0.89	1.89	2.769 (2)	172
N4—H4*C*···O3^iii^	0.89	1.84	2.728 (2)	176
N5—H5*A*···O8^v^	0.89	1.83	2.701 (2)	165
N5—H5*B*···O2^i^	0.89	1.93	2.762 (2)	156
N5—H5*C*···O1	0.89	2.09	2.945 (2)	162
N6—H6*A*···O10^vi^	0.89	1.85	2.720 (2)	166
N6—H6*B*···O11	0.89	2.04	2.900 (2)	163
N6—H6*C*···O12^iii^	0.89	1.92	2.744 (2)	153

Symmetry codes: (i) *x*−1, *y*, *z*; (ii) −*x*+1/2, *y*+1/2, −*z*+1/2; (iii) *x*+1, *y*, *z*; (iv) *x*, *y*−1, *z*; (v) *x*, *y*+1, *z*; (vi) −*x*+3/2, *y*−1/2, −*z*+1/2.
