# Supplementary figures and images for: Crystal structure of bis­(allyl­ammonium) oxalate
Source: Acta Crystallogr Sect E Struct Rep Online. 2014 Nov 5;70(Pt 12):o1229–30. doi: 10.1107/S1600536814023617 (PMC4257458; doi:10.1107/S1600536814023617)

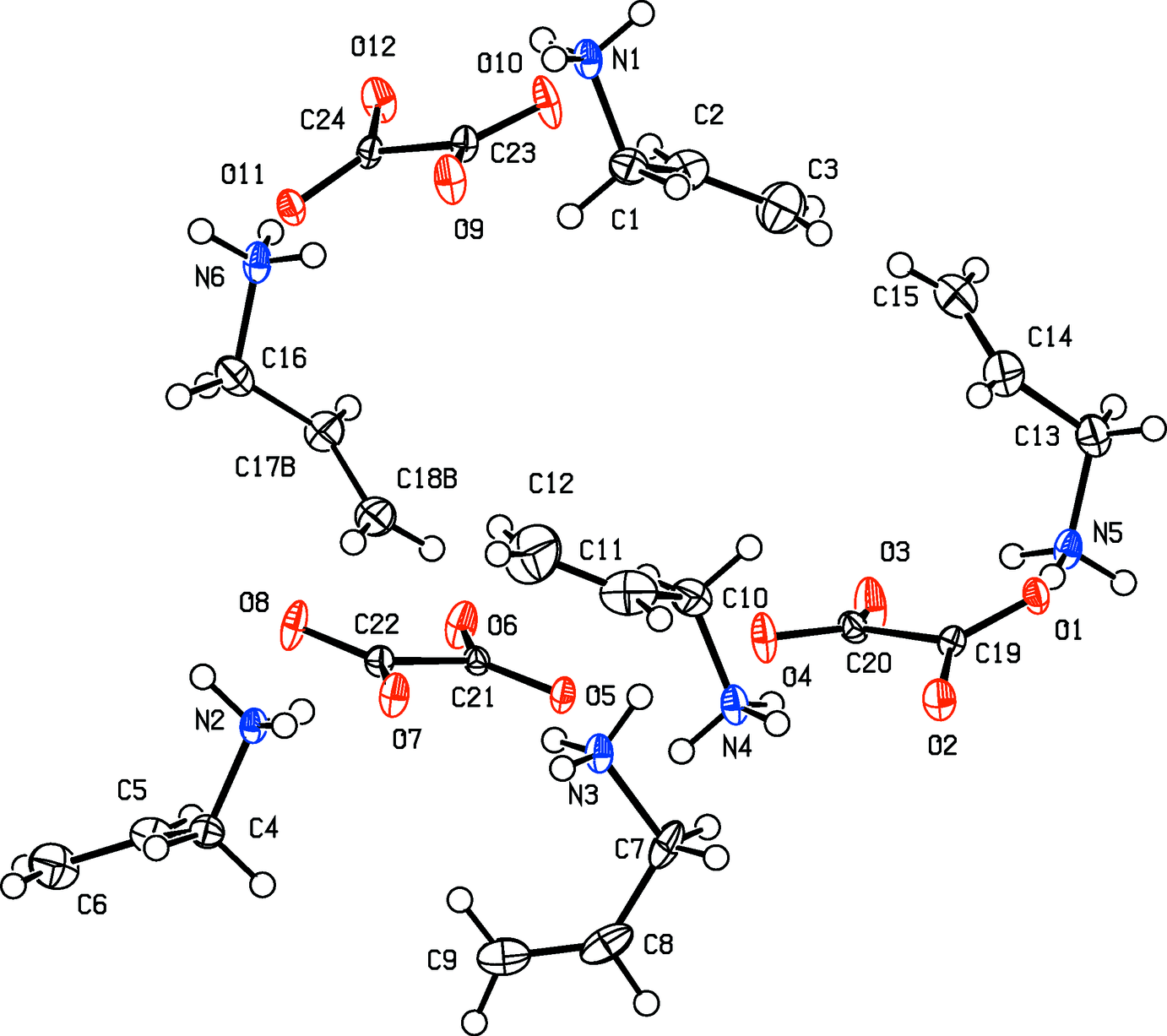

Supplement: Supplementary file 4 [file e-70-o1229-fig1.tif]

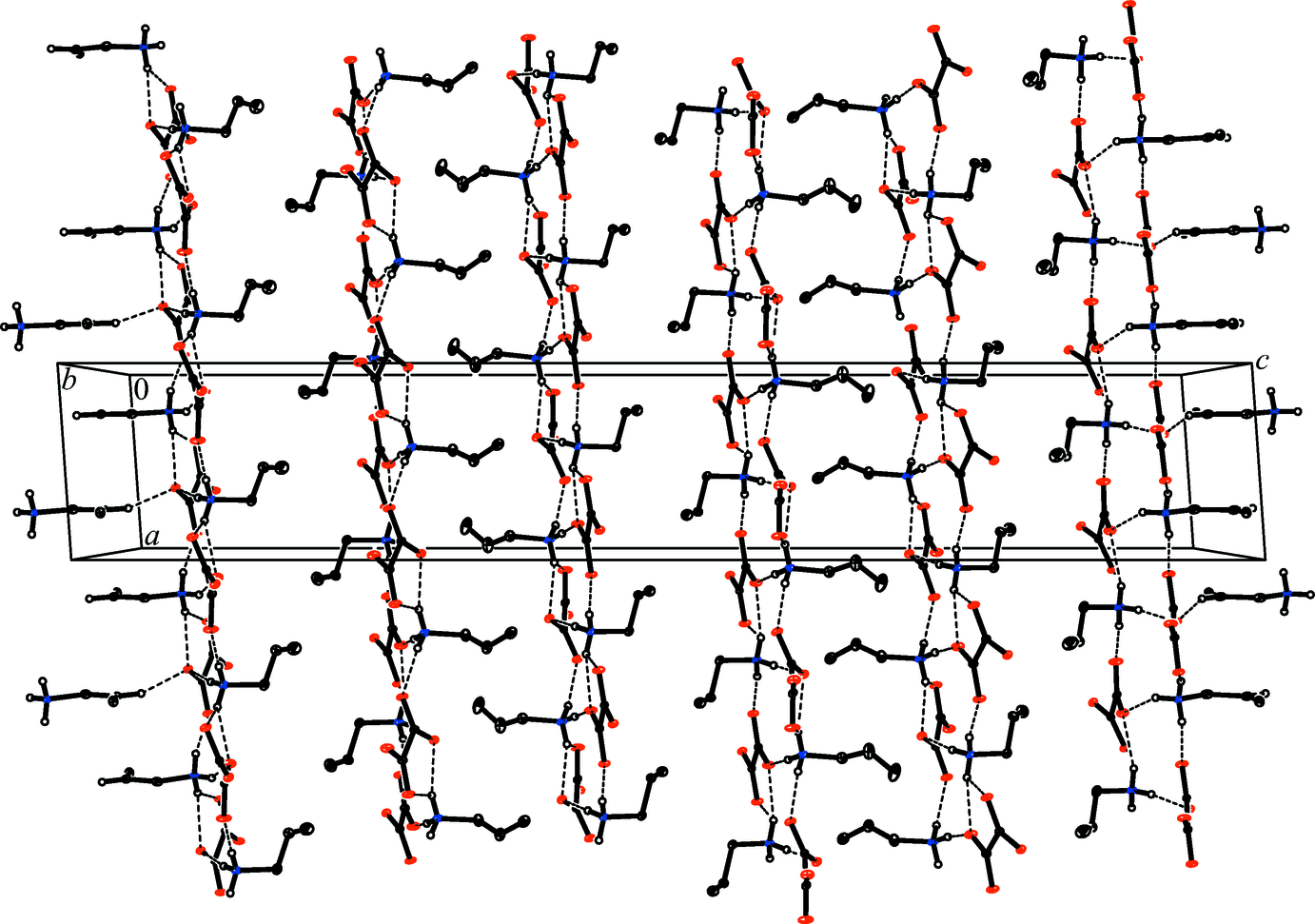

Supplement: Supplementary file 5 [file e-70-o1229-fig2.tif]
